# Association between housing environment and depressive symptoms among older people: a multidimensional assessment

**DOI:** 10.1186/s12877-021-02207-9

**Published:** 2021-04-17

**Authors:** Yuan Chen, Ping Yu Cui, Yi Yang Pan, Ya Xing Li, Nuremaguli Waili, Ying Li

**Affiliations:** Department of Social Medicine, School of Public Health, Zhejiang University, 866 Yu-hang-tang Road, Zhejiang, 310058 Hangzhou China

**Keywords:** Depressive symptoms, Housing environment, Multidimensional assessment, Cumulative logistic regression analysis, Cross-sectional study

## Abstract

**Background:**

Depression is a common mental disorder among older people. This study aimed to assess the association between housing environment factors and depressive symptoms among older people using a multidimensional assessment method.

**Methods:**

The study *uses* a population-based cross-sectional design*.* A total of 950 participants aged ≥ 60 years were selected using a complex multistage sampling design from 22 locations in China. All data were collected using questionnaires by face-to-face interviews. A total of 938 participants were included in the analysis, and 17.1% of males and 23.1% of females were identified as having depressive symptoms. The depressive symptoms were assessed using the 15-item Geriatric Depression Scale. The housing environment was assessed on the basis of four dimensions: physical, social, psychological, and cognition and physical function. Cumulative logistic regression analysis was used to evaluate the association between housing environment and depressive symptoms.

**Results:**

The Cochran–Armitage trend test showed that the depressive symptom scores were linearly negatively associated with self-assessed housing environment, living arrangement, life satisfaction, and other physical environment factors and linearly positively associated with cognitive and physical function scores. The results of cumulative logistic regression analysis showed that the housing environment was significantly associated with depressive symptoms. The participants’ self-assessed housing environment was strongly associated with the levels of depressive symptom scores, and the odds ratio was *3.47 (*95% CI, *1.14*–*10.82, P* = 0.003*)*.

**Conclusion:**

The housing environment was significantly associated with depressive symptoms. Our results suggest that multi-dimensional assessment in the housing environment may be an effective way to develop intervention strategies of depressive symptoms among older people.

## Background

With the growing population of older people worldwide, health problems and challenges for individuals, society, and public health systems are increasing. A common concern is that the prevalence of depression among older people increases with increasing life expectancy [1, 2]. The prevalence ranges from 6 to 80% in different countries and regions of around the world [3, 4].

As an important mental health problem, depression is a major cause of global disability and suicide and may be associated with cardiovascular diseases and mortality through a complex and potential mechanism of biological crosstalk [5–7]. Severe depression impairs the lives of older people more than serious medical illnesses, and treatment of depression has the potential to improve their lives in spite of other medical comorbidities [8, 9].

The causes of depression are complex and incompletely understood. Depression is often considered as a result of medical illness, but social, behavioral, and psychological factors are commonly considered as important risk factors for depressive disorders [10–12]. Many studies have consistently found that behavioral and psychosocial factors, such as alcohol and substance abuse, smoking, sleep disturbance, physical inactivity, unhealthy eating habits, and stressful events, and sociodemographic factors, such as low income, unemployment, low education level, and low social support, are mostly associated with an increased risk of depressive behaviors [13–16]. In addition, chronic diseases, bereavement, retirement, social isolation, and loss of income are major risk factors for depression among older people [17].

Recently, the association between environmental factors and depression has attracted increasing interest. Previous studies have shown that physical, social, and psychological environments may affect an individual’s mental health and also suggested that the environment may play a particularly important role in the mental health of older adults compared with younger adults [18]. Moreover, the number of care homes has decreased as more older people are choosing to live at home. These people expect to spend as much time at home as possible in their last years. As a result, the housing environment has become more important to them.

Our understanding of the health effects related to the housing environment has evolved over the past two decades. During this period, the focus of studies on the housing environment has shifted from indoor air constituents such as fuel use to accessible housing [19]. Older people living in accessible homes and independently performing daily activities were found to have better well-being and suffer less from depressive symptoms [20]. However, studies focusing on depressive symptoms and the housing environment generally pay little attention to other environment factors or have weak research designs, often lacking control for confounding variables. In addition, previous studies have evaluated the association between housing environmental exposure and the risk of mental disorders in the general population. Studies have shown that older people spend 90% of their time indoors and have a longer period of housing environmental exposure compared with the general population. Therefore, the previous evaluation results might have underevaluated the situation of older people.

However, the pathogenesis of depression in older people is complex and not fully clear. Environmental psychology is a field of applied social psychology that studies the relationship between environment and human psychology and behavior. According to systematic theory, the natural environment and social environment are unified, and both have an important influence on behavior. Therefore, we hypothesized that depressive symptoms are related to the housing environment. The present study aimed to assess the association between housing environment factors and depressive symptoms among older people by using a multidimensional assessment method.

## Methods

### Sampling procedure

The study was based on the “Accessibility Evaluation of Health-Related Resources for the Elderly” project, a cross-sectional design that aimed to assess the association between housing environment and depressive symptoms among older people. A total of 950 general residents aged ≥ 60 years were selected using a multistage sampling design. Sampling was conducted in 22 locations of four provinces (Zhejiang, Heilongjiang, Xinjiang, and Sichuan) in China. We excluded subjects who were unable to complete the questionnaire. All participants provided written informed consent before participation in this study. The study was approved by the institutional review board at the School of Medicine, Zhejiang University.

### Sample description

The characteristics of the study participants by sex are described in Table 1. Among them, 41.7% were male, and 58.3% were female. The participants had an average age of 68.5 years, and 38.0% of the participants were over 70 years of age. Approximately 24.1% of women reported that they had a lower level of education and completed only 6 years or less of education. Men reported higher frequency of smoking and drinking than women, and women reported that they had higher levels of physical activity than men. More than 67.0% of participants reported that they had one or more chronic diseases. Approximately 17.1% of men and 23.1% of women had a depressive symptom score greater than or equal to 5 points.

**Table 1:** Characteristics of study participants by gender in the study

Variable categories	Men (*N* = 391)		Women (*N* = 547)		All (*N* = 938)	
	n %		n %		n %	
Age (yr.)						
60–69	233	(59.6)	346	(63.2)	579	(61.7)
70–79	135	(34.5)	157	(28.7)	292	(31.1)
≥ 80	23	(5.9)	44	(8.1)	67	(7.2)
Education levels (yr.)						
0–6	45	(11.5)	132	(24.1)	177	(18.9)
7–9	166	(42.5)	191	(34.9)	357	(38.1)
10–12	98	(25.1)	120	(22.0)	218	(23.2)
13+	82	(20.9)	104	(19.0)	186	(19.8)
Individual income						
¥0 to 1999	235	(60.1)	319	(58.3)	554	(59.1)
¥2000 to 3999	88	(22.5)	155	(28.3)	243	(25.9)
¥4000 and Over	68	(17.4)	73	(13.4)	141	(15.0)
Marital status						
Married	354	(90.5)	400	(73.1)	754	(80.4)
Non-married	37	(9.5)	147	(26.9)	184	(19.6)
Smoking status						
Yes	120	(30.7)	8	(1.5)	128	(13.7)
No	271	(69.3)	539	(98.5)	810	(86.3)
Alcohol use						
Yes	151	(38.6)	41	(7.5)	192	(20.5)
No	240	(61.4)	506	(92.5)	746	(79.5)
Regular physical activity						
Yes	136	(34.8)	239	(43.7)	375	(40.0)
No	255	(65.2)	308	(56.3)	563	(60.0)
Chronic disease status						
Yes	263	(67.3)	365	(66.7)	628	(67.0)
No	128	(32.7)	182	(33.3)	310	(33.0)
Time before falling asleep (min)						
≤ 10	124	(31.7)	157	(28.7)	281	(30.0)
11–29	59	(15.1)	105	(19.2)	164	(17.5)
30–59	134	(34.3)	171	(31.3)	305	(32.5)
≥ 60	74	(18.9)	114	(20.8)	188	(20.0)
Depression score (points)						
< 5	324	(82.9)	421	(76.9)	745	(79.4)
5–9	54	(13.8)	107	(19.6)	161	(17.2)
≥ 10	13	(3.3)	19	(3.5)	32	(3.4)

### Information collection

#### Questionnaires

Data were collected through questionnaires via face-to-face interviews. The interviews were conducted by investigators in the home and community. The questionnaires consisted of nine parts, including 428 items. The main contents included the following aspects: demographic characteristics, general health status and behavior habits, medical resources, community health service resources, psychological resources, environmental resources, and activities of daily living assessment. The interviews took approximately 45–60 min to complete for most of the participants. Content validity of the questionnaire is established through a panel of six expert judges. Data were obtained from a Likert-type rating scale. The content validity index on the levels of items were from 0.90 to 1.00, the content validity index of average scale level was 0.98 and the content validity ratios were from 0.86 to 1.00. Before the formal investigation, we produced a preliminary survey to verify the validity of the questionnaire. We computed the total sample size required for this work based on the empirical study and the events per variable method.

The general characteristics of participants, including age, gender, ethnicity, income, education level, self-reported chronic disease status, disease history, daily habits, and physical activity level, were recorded. Environmental resources were identified from housing and surrounding housing environments. The exposure to housing environment was assessed in terms of four aspects: physical, social, psychological, and cognition and physical function.

#### Housing environment

The physical environment of the house was assessed by using a self-assessment instrument. The psychological and cognition environment and social environment were measured using two instruments. These instruments have been proven to be valid and reliable in previous study [21–23]. The physical environment was measured using the following questions: “Is there enough sunlight in your house?” “Is your house well ventilated?” The responses were categorized as “yes” or “no.” “How many square meters is your house?” “How long have you lived in your present house?” The participants were also asked about their use of shower and heating.

The social attributes of the housing environment were measured using the living arrangement and neighborhood communication. “Who do you live with right now” as the instrument of living arrangement contained seven items: living alone, living with spouse, living with children, living with grandchildren, living with parents, living with caregiver, and others. The participants were asked how many times they visited their neighbors in the past week. The responses were categorized into four levels: “none,” “once a week,” “2–3 times a week,” and “more than 3 times a week.”

The psychological attribute of the housing environment was assessed using the following question: “Are you satisfied with your daily life?” The responses were categorized as “yes” or “no.” The Dementia Assessment Sheet for Community-based Integrated Care System-21 items (DASC-21) was used to assess the characteristics of cognition and physical function for the housing environment. The instrument contained 21 questions, and the responses were divided into four levels. Each level was scored from 1 to 4. The total score was 84 points, and the cut-off point of 31 points was used to identify cognition and physical function impairment.

Two questions were used to assess the self-assessed housing environment and surrounding housing environment: “Do you think that your house is clean and comfortable?” and “Do you think that the surrounding housing environment is clean and comfortable?” Exposure to the surrounding housing environment was also measured using the following questions: “Is there a factory or waste treatment plant near your house?” “Are factors such as air pollution, noise, low air pressure, humidity, and dryness present around your housing?” The responses were categorized as “yes” or “no.”

#### Depressive symptom measurement

Depressive symptoms were assessed using the 15-item Geriatric Depression Scale (GDS-15). The GDS-15 was designed as a self- or interviewer-administered screening instrument and consists of 15 questions addressing various depressive symptoms. The 15 questions have been described in detail in a previous study [24]. The optimal cut-off point to identify depressive symptoms was 5 points; ≥10 points indicated moderate to severe depressive symptoms, 5–9 points indicated mild depressive symptoms, and 0–4 points indicated no depressive symptoms among older people [25].

#### Measurement of other factors

Social support was assessed using the Chinese version of the Older Americans’ Resources and Services questionnaire. The participants’ personality characteristics were assessed using the Eysenck Personality Questionnaire (EPQ).

### Statistical analysis

Statistical analysis was restricted to the 938 participants with complete questionnaires and depressive symptom assessment data. Descriptive statistics were used to describe the general characteristics of the study participants.

On the basis of the cut-off values of GDS-15 for distinguishing the degree of depressive symptoms, depressive symptoms as a dependent variable were classified into three categories in accordance with the depressive symptom scores of participants. Chi-square tests were used to perform univariate analysis with the housing environment as an independent categorical variable. Univariate analysis was performed to assess the association between the exposure of housing and surrounding environment and depressive symptoms. The Cochran–Armitage test was used to assess the linear association of depressive symptoms with the levels of exposure to housing and surrounding environment.

We used *two separate* cumulative logistic regression models to evaluate the association between depressive symptoms and housing environment. Depressive symptom scores were treated as rank variables and added to the cumulative logistic regression model as dependent variables. If the participant’s depressive symptom score was less than 5 points, it was expressed as “0” in the dependent variable of the logistic regression model; otherwise, “1” if their score is 5 to 9 points and “2” if their score is greater or equal to 10 points. In model 1, the variables of the physical environment and the variable of self-assessed housing environment as independent variable were added to the model through a stepwise method. The model was adjusted for the levels of social support, time before falling asleep, and EPQ score. Model 2 included the association of housing environment variables with social, psychological, cognition, and physical function. Both models were also adjusted for age, gender, region, education level, physical activity, income, chronic disease status, eating habits, mobile phone use, sleep time, and income satisfaction.

Binary logistic regression was used to identify the association of factors with the self-assessed housing environment. The significance level for all analyses was set at *P* < 0.05. All analyses were performed using SAS for Windows (version 9.4).

## Results

Table 2 shows the characteristics of housing and surrounding environment categorized on the basis of depressive symptom scores. The results of univariate analysis showed that environmental exposure to housing and surrounding risk factors was associated with depressive symptoms.

**Table 2:** The depression scores by participants reported the characteristic of housing environment

Variable categories	Depression score (points)						*P* value	*P* for trend
	< 5		5–9		≥ 10			
	n	%	n	%	n	%		
Exposure to housing environment								
Adequate sunshine								
Yes	677	(98.2)	180	(91.4)	46	(88.5)	< 0.001	< 0.001
No	12	(1.8)	17	(8.6)	6	(11.5)		
Good ventilated								
Yes	679	(98.5)	186	(94.4)	46	(88.5)	< 0.001	< 0.001
No	10	(1.5)	11	(5.6)	6	(11.5)		
Living areas (m^2^)								
≤ 80	209	(30.3)	69	(35.0)	28	(53.9)	0.001	0.001
> 80	480	(69.7)	128	(65.0)	24	(46.1)		
Residence times (yr.)								
≤ 30	320	(46.4)	65	(33.0)	19	(36.5)	0.002	0.002
> 30	369	(53.6)	132	(67.0)	33	(63.5)		
Working shower								
Yes	598	(86.8)	144	(73.1)	45	(86.5)	< 0.001	0.004
No	91	(13.2)	53	(26.9)	7	(13.5)		
Living arrangement								
Living alone	546	(79.3)	75	(38.1)	13	(25.0)	< 0.001	< 0.001
Living with family or other	143	(20.7)	122	(61.9)	39	(75.0)		
Neighborhood communication								
None	253	(36.7)	81	(41.1)	29	(55.8)	0.018	0.008
At least once a week	436	(63.3)	116	(58.9)	23	(44.2)		
Life satisfaction								
Yes	677	(98.3)	157	(79.7)	26	(50.0)	< 0.001	< 0.001
No	12	(1.7)	40	(20.3)	26	(50.0)		
Cognitive and physical function (points)								
≤ 30	567	(82.3)	83	(42.1)	16	(30.8)	< 0.001	< 0.001
> 30	122	(17.7)	114	(57.9)	36	(69.2)		
Clean and comfortable								
Yes	685	(99.4)	184	(93.4)	38	(73.1)	< 0.001	< 0.001
No	4	(0.6)	13	(6.6)	14	(26.9)		
Exposure to surrounding housing environment								
Air pollution or noise								
Yes	65	(9.4)	32	(16.2)	11	(21.2)	0.002	< 0.001
No	624	(90.6)	165	(83.8)	41	(78.8)		
Clean and comfortable								
Yes	647	(93.9)	164	(83.3)	43	(82.7)	< 0.001	< 0.001
No	42	(6.1)	33	(16.7)	9	(17.3)		

For the self-assessed housing environment, 0.6, 6.6, and 26.9% of participants with depressive symptom scores of < 5, 5–9, and ≥ 10 points responded with “no” when asked “Do you think that your house is clean and comfortable?” respectively. For the physical environment, *participants* who *reported* that their house *had* adequate *sunlight* exposure, good ventilation, living areas *of* more than 80 m^2^, and working shower *and who had a* residence time of less than 30 years *had a* significantly lower rate of high depressive symptom scores compared with others. The analysis of social and psychological factors contributing to housing environment showed that *participants* who *lived* alone, had poor neighborhood communication, and had low life satisfaction had high depressive symptom scores. For exposure to the surrounding environment, 6.1, 16.7, and 17.3% of participants with depressive symptom scores of < 5, 5–9, and ≥ 10 points responded with “no” when asked “Do you feel clean and comfortable near your house?” respectively. The participants who answered “no” had the highest percentage in the highest category of depressive symptom score. The participants who reported noise or air pollution near their house had high levels of depressive symptom scores. The results of the Cochran–Armitage test showed that the depressive symptom scores were linearly negatively associated with adequate sunlight, good ventilation, living areas, working shower, living arrangement, life satisfaction, and self-assessed housing environment and linearly positively associated with residence time and cognitive and physical function scores.

The odds ratios (ORs) of the housing environment and other related factors for depressive symptoms by separated cumulative logistic regression models are shown in Table 3. In model 1, the participants’ self-assessed housing environment was strongly negatively associated with levels of depressive symptom scores in the cumulative logistic regression analysis. The OR was 10.75 (95% CI, 4.39–26.31, *P* < 0.001) for participants who responded with “no” when asked “Do you think that your house is clean and comfortable?” compared with those who responded with “yes.” This association was maintained in model 2 after adjusting for *a large number of risk factors, and the OR was 3.47 (*95% CI, *1.14*–*10.82, P* = 0.003*)*. As a physical characteristic of the housing environment, the residence time was positively associated with depressive symptom scores, and the OR was 1.04 (95% CI, 1.01–1.12, *P* = 0.042). According to the stepwise regression model, factors such as social support levels, frequency of contact with children, EPQ score, time before falling asleep, and noise and air pollution around the house were associated with the levels of depressive symptom scores. In model 2, low life satisfaction as a psychological characteristic of the housing environment was associated with depressive symptom scores, and the OR was 5.43 (95% CI, 1.61–6.04, *P* < 0.001). As one attribute of the housing environment, cognitive and physical function was positively associated with depressive symptom scores, and the OR was 1.09 (95% CI, 1.05–1.14, *P* < 0.001). The level of neighborhood communication, a social attribute of the housing environment, was associated with depressive symptom scores, and the OR was 2.01 (95% CI, 1.21–3.36, *P* < 0.001). The model was also adjusted for the use of mobile phones, level of income satisfaction, and housing equipment.

**Table 3:** The odds ratios of housing environment and other related risk factors for depression by cumulative logistic regression models

Variables	Multivariable adjusted			*P* value
	Odd Ratios	95% CI		
Model 1				
Do you think that your house is clean and comfortable (y/n) ^sa^	10.75	4.39	26.31	< 0.001
Air pollution and noise around house (n/y) ^pe^	2.72	1.41	5.25	0.002
High levels of social support (points)	0.81	0.75	0.88	< 0.001
Low frequency of contact with children (weeks)	1.36	1.09	1.70	0.006
Long time before falling asleep (min)	1.58	1.31	1.89	< 0.001
Regular physical activity (n/y)	0.45	0.27	0.72	0.001
High EPQ scores (points)	1.16	1.10	1.22	< 0.001
Model 2				
Do you think that your house is clean and comfortable (y/n) ^sa^	3.47	1.14	10.82	0.003
Long residence times (yr.) ^pe^	1.04	1.01	1.12	0.042
Air pollution and noise around house (n/y) ^pe^	3.32	1.66	8.75	0.002
Low life satisfaction (y/n) ^pse^	5.43	1.61	6.04	< 0.001
Poor cognitive and physical function (points) ^ce^	1.09	1.05	1.14	< 0.001
Poor neighborhood communication (times) ^se^	2.01	1.21	3.36	< 0.001
Living alone ^se^	1.44	0.60	3.49	0.340
High levels of social support (points)	0.82	0.73	0.92	< 0.001
Low frequency of contact with children (weeks)	1.35	1.02	1.77	0.035
High EPQ scores (points)	1.16	1.10	1.24	< 0.001
Long time before falling asleep (min)	1.45	1.16	1.70	< 0.001

sa: self-assessed housing environment

pe: physical attributes of the housing environment

se: social attributes of the housing environment

pse: psychological attribute of housing environment

ce: cognition attribute of housing environment

The results of the analysis of related factors for self-assessed housing environment are shown in Table 4. The self-assessed housing environment conditions were significantly associated with the following environmental factors: good ventilation, working shower, clean and comfortable surrounding environment, levels of cognitive and physical function, social support levels, and time before falling asleep.

**Table 4:** The effect factors association with the self-assessed housing environment

Variables	Multivariable adjusted			*P* value
	Odds Ratios	95% CI		
Good ventilated (n/y)	8.28	1.86	12.70	0.006
Working shower (n/y)	3.82	1.30	11.28	< 0.015
Long time before falling asleep (min)	0.53	0.34	0.82	0.004
High levels of social support (points)	1.30	1.13	1.51	< 0.001
Poor cognitive and physical function (points)	0.11	0.04	0.29	< 0.001
Clean and comfortable surrounding housing environmental (y/n)	4.12	1.41	12.05	0.009

Models adjusted for age, gender, region, education level, regular physical activity, income, chronic disease status, habit of food intake, mobile phone use, sleep duration and income satisfaction

Participants who responded with “yes” when asked “Do you think that your house is clean and comfortable?” were associated with good ventilated (OR 8.28 [95% CI, 1.86–12.70]), working shower (OR 3.82 [95% CI, 1.30–11.28]), clean and comfortable surrounding environment (OR 4.12 [95% CI, 1.41–12.05]), poor cognitive and physical function (OR 9.45 [95% CI, 3.44–25.97]), and high levels of social support (OR 1.30 [95% CI, 1.13–1.51]).

## Discussion

In the present study, we found that the housing environment was significantly associated with depressive symptoms among older people. We further observed that the housing environment was associated with depressive symptoms based on four attributes: physical, social, psychological, and cognition and physical function. In addition, the self-assessed housing environment was more associated with depressive symptoms than the housing environment based on the four attributes. *C*lean and comfortable housing environments were strongly negatively associated with depressive symptoms after adjusting for personality characteristics and a number of potential confounding factors. The factors associated with the self-assessed housing environment were identified in the study. Good ventilation, working shower, clean and comfortable surrounding environment, cognitive and physical function, and level of social support were mainly associated with the self-assessed housing environment. In our study, ventilation was significantly associated with the self-assessed housing environment.

### Initial research on the housing environment

Over the past 30 years, several studies have investigated the housing environment in different related fields. The study focus of the housing environment is also changing with the continuous development of society and our understanding of the association between health and the environment. In the past, the housing environment is generally understood as the indoor environment. Studies of housing environment focused on physical and chemical factors such as indoor air constituents, temperature, and humidity [26]. These studies showed that the pollutants in the indoor environment may come from fuel combustion during cooking, heating, smoking, and construction, and ventilation is necessary to reduce concentrations of pollutants generated indoors [27].

### Physical function and housing environment

Due to the progression of medical technology, the life expectancy has increased. Moreover, the number of physically challenged and disabled older people is also increasing. Falls have become a public health problem for older people. The focus of study on the housing environment has gradually shifted from physical attributes to fall prevention and barrier-free housing design [28, 29]. Some studies have explored how to improve the housing environment to help older people adapt to the decline of functional ability, thereby reducing the prevalence of anxiety and depression and maintaining happiness and independence in daily life. However, there is limited evidence to evaluate the association between adaptability of individual physical function for housing environment and depression [30, 31]. The results of the present study showed that the level of physical function of participants was positively associated with their self-assessed housing environment, and those who reported that their houses were comfortable and tidy had low depressive symptom scores.

### Social, psychological, and housing environment

With the rapid development of society and the economy, relationship problems among family members may occur. An increasing number of researchers are interested in studying the social attributes of the housing environment caused by the number of empty nesters. In addition, the number of older people living alone is increasing. Previous studies have shown that elderly individuals who live alone are associated with an increased risk for depressive symptoms [23, 32–34]. In the present study, we further found that adequate social support can improve the increased risk of depressive symptoms due to living arrangements as the living arrangement was no longer statistically significant in the model. In addition, the study on the social attributes of the housing environment includes the effect of neighborhood communication and economic differences on mental health outcomes [35–37]. Recently, studies on residential satisfaction and well-being, which are psychological attributes of the housing environment, have been widely conducted [38, 39]. However, some important risk factors, such as personality factor, are rarely considered. In the present study, we used the EPQ scale to assess the personality characteristics of participants, and the association between housing environment factors and depressive symptoms was adjusted using the EPQ score to remove the effect of the important risk factor. However, previous studies have not comprehensively explored the association between multiple attributes of the housing environment and depressive symptoms among older people. Previous analyses lacked evaluation models to make an objective assessment of depressive symptom risks. Evidence on how to improve the housing environment of older people, promote mental health, and reduce the prevalence of depressive symptoms is also lacking.

### Conduct studies on the housing environment

To our knowledge, no studies have used cognitive function as an attribute of the housing environment to evaluate the association between housing environment and depressive symptoms. In addition, whether individuals have a correct understanding and perception for the housing environment has not been considered when evaluating the association between housing environment and depressive symptoms. Our results showed that the cognitive function level is positively associated with the perceived assessment of the housing environment. Individuals with high cognitive level have high adaptability and high evaluation for their living housing environment, and those with high evaluation for their living housing environment are associated with lower risk of depressive symptoms. In this study, we not only assessed the association between housing environment and depressive symptoms in terms of physical, social, and psychological attributes, but also assessed the association between depressive symptoms and housing environment in terms of cognitive function.

### Suggestions

With aging, older people become more vulnerable to environmental challenges and depression. More older people will spend their time in their own houses. The housing environment is particularly important for the well-being of older people, especially mental health. With the large amount of time spent in the house, the housing environment is a potential important factor for increasing and improving the prevalence of depressive symptoms. Many countries are planning and preparing to implement housing assistance programs around the world [40, 41]. However, there is no reliable evidence on how to reduce depressive symptoms in older people by improving the housing environment. Apartments or home for the aged is an important location for older people to live in their later years. The housing environment can promote the mental health of older people, but it can also lead to severe depressive symptoms. Our results suggest that to improve the housing environment and develop intervention strategies of depressive symptoms, we must consider the attributes of the housing environment and adopt comprehensive intervention strategies centered on the physical environment, social environment, psychological environment, and cognitive and physical functions, so that older people can successfully avoid depressive symptoms and live at home healthily. We can use accessible resources to improve the adaptability between individuals and housing environment.

### Limitations

The present study has one major limitation that must be addressed. This was a cross-sectional design. The associations between depressive symptoms and housing environment from multiple dimensions were observed, but the causality could not be discussed. This complex and changing trend of environmental factors and the risk of depressive symptoms over time cannot be evaluated and observed. In addition, compared with the measured housing environment, the self-assessed housing environment is more associated to depressive symptoms; therefore, the negative assessment could be a result of the depressive state of mind, which can lead to reporting bias.

## Conclusions

The housing environment has an important impact on depressive symptoms. This study indicates that depressive symptoms are significantly associated with the housing environment from several aspects, including physical, social, and psychological. Correct cognition of the housing environment and adaptability of physical function to the housing environment as an attribute of the housing environment are also associated with depressive symptoms. In addition, although our study fully considered the housing environment variables and important risk factors from multiple dimensions, we could not obtain a large enough sample size to evaluate the interactions between several dimensions of housing environment and depressive symptoms. This study suggests that the association between housing environment and depression symptoms is complex. A longitudinal study design is needed to confirm the causality of these factors in the future.

## Data Availability

The datasets used and/or analyzed during the current study are available from the corresponding author on reasonable request.

## Abbreviations

DASC-21: Dementia Assessment Sheet for Community-based Integrated Care System-21 items
OARS: Older American Resources and Services
EPQ: Eysenck Personality Questionnaire
GDS-15: 15-item Geriatric Depression Scale
ORs: Odds ratios
CI: Confidence interval
